# Does digital X-ray radiogrammetry have a role in identifying patients at increased risk for joint destruction in early rheumatoid arthritis?

**DOI:** 10.1186/ar4058

**Published:** 2012-10-15

**Authors:** Kristina Forslind, Johan Kälvesten, Ingiäld Hafström, Björn Svensson

**Affiliations:** 1 Section of Rheumatology, Department of Medicine, Helsingborg Hospital, Södra Vallgatan 5, 25187 Helsingborg, Sweden; 2 Section of Rheumatology, Institution of Clinical Science, University Hospital, Klinikgatan 15, 22242 Lund, Sweden; 3 Section of Radiology, Department of Medicine and Health Sciences, University Hospital, 58185 Linköping, Sweden; 4 Center for Medical Image Science and Visualization (CMIV), Linköping University, 58183 Linköping, Sweden; 5 Sectra Imtec AB, Teknikringen 20, 583 30 Linköping, Sweden; 6 Rheumatology Unit, Karolinska Institutet, Karolinska University Hospital, 14186 Huddinge, Sweden

## Abstract

**Introduction:**

The aim of this study was to investigate the role of hand bone mineral density (BMD) loss analyzed with digital X-ray radiogrammetry (DXR) in early rheumatoid arthritis (RA) as a predictor for progression of joint damage.

**Methods:**

In 379 patients with early RA, baseline and one-year hand BMD was measured with DXR and the hand bone loss (HBL) was analyzed using the smallest detectable change (HBLsdc) and tertiles (HBLtertiles). Joint damage in hands and feet were scored according to the Sharp van der Heijde (SHS) method at baseline and at one, two, five and eight years. At the same time-points Disease Activity Score (DAS28) was calculated and functional disability assessed. Rheumatoid factor (RF) and antibodies against cyclic citrullinated peptides (anti-CCP) were analyzed at baseline.

**Results:**

Sixty-six percent of the patients had hand BMD loss in the first year of RA determined by HBLsdc and 65% by HBLtertiles. Radiographic progression after two, five and eight years was associated with hand bone loss defined by HBLsdc. By HBLtertiles there were significant associations at all time-points except at eight years. The change in DXR at one year (ChDXR_1yr_) correlated significantly and inversely with the change in SHS (ChSHS) at two, five and eight years. Multivariate analysis showed that only change in SHS during the first year and the presence of anti-CCP were independent predictors of long-term progressive joint damage. If radiographic scores were not included, DXR-BMD loss was an independent predictor. Patients with great bone loss by HBLtertiles had significantly more often high disease activity after two years. However, neither bone loss by HBLsdc or HBLtertiles nor by ChDXR_1yr _was an independent predictor of remission after two, five and eight years.

**Conclusions:**

This study confirms previous reports of an association of decrease in DXR-BMD during the first disease year with progression of radiographic joint damage over an extended period of time. This association was independent in a regression model only when radiological findings were excluded suggesting a possible predictive role of DXR-BMD in clinical practice when radiographic evaluation is not available. However, further studies are required before this can be established.

## Introduction

Rheumatoid arthritis (RA) is a progressive autoimmune disease characterized by inflammatory activity in the joints, synovial sheets of tendons and bursae as well as extra-articular manifestations such as vasculitis and serositis [1-3]. The increased amount of pro-inflammatory cytokines mediates osteoclast activation, which in turn provokes joint destruction [4].

Both the disease and its drug treatment can cause systemic bone loss and also a periarticular disease-related osteoporosis [5]. The periarticular bone loss in hands is an early feature of RA and may precede erosions [6,7]. As periarticular demineralization and joint damage are both related to imbalance in osteoclast and osteoblast activity, measures of hand bone loss may be a marker for active deterioration of bone and a predictor of subsequent erosive joint damage.

Radiogrammetry was introduced in the sixties for assessment of skeletal status, using various measures of the cortical bone on conventional hand radiographs. The diaphysis of the second metacarpal of the hand was often selected for radiogrammetry. Measurements of the total and medullar width of the bone can be used to calculate different indices and to consecutively quantify changes of cortical bone [8].

The availability of digital images provides the opportunity for quantitative measurements of radio-geometric features and offers a refinement for radiogrammetry [9,10]. Digital X-ray radiogrammetry (DXR) is a technique that uses automated image analyses of standard hand radiographs, either in digital or conventional analog format, to estimate bone mineral density (DXR-BMD) [8,11,12]. DXR was first introduced into clinical practice in 1999 for osteoporosis assessment. Several studies have shown that this technique has a potential to predict progressive joint damage in RA [13-15].

The heterogeneity of the RA disease and the need for rapid adjustment of disease management puts high requirements on markers for disease progress. The currently available predictors for poor outcome are not perfect and there is a need for improved decision support. The aim of this study was to investigate the potential of hand BMD loss analyzed with DXR to predict progression of joint damage in patients with early RA followed for up to eight years.

## Materials and methods

### Patients

BARFOT (Better Anti-Rheumatic FarmakOTherapy) is a multicenter observational study (six centers in southern Sweden) of patients with recent (disease duration < one year) onset RA satisfying the 1987 American College of Rheumatology (ACR) classification criteria [6]. Eight hundred and thirty nine (839) patients were consecutively enrolled into the BARFOT study [16] between 1993 and 1999. The patients were between 18 and 80 years of age. The present study comprised the 379 patients who had radiographs available at baseline and one-year follow up, which were suitable for DXR-BMD measurement. Of those with accessible and readable radiographs, four patients were excluded due to improper positioning of the hand. During the observation period, patients were treated according to clinical judgment by their rheumatologist, except for 166 patients participating in a randomized low-dose glucocorticoid study [17].

### Radiographic evaluation

Radiographs of hands and feet were taken at study entry (baseline) and after one, two, five and eight years. At two years radiographs were available for 355 patients, at five years for 288 patients and at eight years for 240 patients. *Radiographic damage *was scored according to the Sharp van der Heijde score (SHS) [18] which includes hands and feet. A total SHS range 0 to 448, consisting of erosion score (E score) range 0 to 280 and joint space narrowing score (JSN score) range 0 to 168. An increase of 1 unit means one new pathologic change - E or JSN. Two trained readers, who were blind for treatment and clinical data, read the films in chronological order.

*Radiographic progression *was defined as a SHS above the smallest detectable change (sdc), which was 5.8, calculated by the formula described by Bruynesteyn *et al. *[19].

### Hand BMD measurements

BMD of the hands was measured by applying DXR, at baseline and after one year, to the same radiographs of hands that were used for radiographic scoring. The analogue X-ray films were digitized using a Vidar Diagnostic Pro plus, 300 dpi, 12 bit (VIDAR Systems Corp., Herndon, VA, USA). DXR-BMD was measured on the digitized images by dxr-online (Sectra, Linköping, Sweden). DXR is a computerized version of the traditional technique of radiogrammetry [20]. The technique has been described in detail previously [8,21]. In short, this method provides a BMD estimate in g/cm² based on an automated analysis of the geometry and texture of the cortical bone of the three middle metacarpals.

When radiographs for both hands were available, the mean BMD was used in the analyses to maximize accuracy of the BMD loss measurement.

#### Hand bone loss

Hand bone loss (HBL) was defined as *present or not present *if the one year change in DXR-BMD was more than 0.0048 g/cm² (4.8 mg/cm²), the smallest detectable change (sdc) [21] or not (HBLsdc) and as *great, moderate or no/small *if the one year change in DXR-BMD was within the upper, medium or lower tertiles (HBLtertiles). Changes in HBL are calculated in relation to baseline values.

### Clinical evaluations

Clinical assessments were performed at baseline, three and six months, and one, two, five and eight years. Disease activity was assessed by the Disease Activity Score calculated on 28 joints (DAS28) [22]. High disease activity is defined as a DAS28 >5.1, moderate >3.2 ≤5.1 and low ≤3.2 [23]. The European League against Rheumatism (EULAR) definition of remission, a DAS28 <2.6, was used [24]. Acute phase reactions were measured by erythrocyte sedimentation rate (ESR, mm/h) and C-reactive protein (CRP, mg/L), according to standard laboratory methods. Patients' estimated general health (GH) was assessed by a 0 to 100 mm horizontal visual analogue scale (VAS) where 0 is best and 100 worst.

Rheumatoid factor (RF) and antibodies against cyclic citrullinated peptides (anti-CCP) were analyzed at baseline. Functional disability was assessed using the Swedish version of the Stanford Health Assessment Questionnaire (HAQ) [25]. The HAQ score ranges from 0 to 3, where a higher score indicates a higher degree of disability.

### Statistics

Statistical analyses were performed using SPSS version 17.0 statistical software. All significance tests were two-tailed and conducted at the 0.05 significance level. To test the differences between groups, the Mann-Whitney U-test or the independent t-test was used for continuous variables, and the chi^2^-test for proportions. The Wilcoxon matched pairs test was used to compare changes of a variable over time. Spearman's correlation test was used to assess the relationships between two continuous variables.

The inter- and intra-observer reliability was assessed by the intra-class correlation coefficient (ICC) (absolute agreement, two-way mixed model) for status and change scores at baseline and two years.

Univariate analyses were performed by score tests and multiple logistic regression analyses were performed including variables with a statistical significance in the score test of p < 0.1.

### Ethics committees

All patients gave their informed consent and the ethical committees in Göteborg: Gbg Ö 282-01; Lund: LU 398-01; Linköping: LI 01-263; Karolinska Institutet: KI 02-075 approved the study.

## Results

### Baseline characteristics of the patients

Demographic and clinical characteristics at baseline for the participating 379 patients are shown in Table 1. Fifty-seven percent of the patients had no erosions at baseline.

**Table 1:** Demographic and baseline clinical characteristics for the 379 patients.

		Mean (SD)	Number (%)
Age at inclusion, years		57 (15)	
Disease duration, months		6.3 (3.2)	
Women			241 (64)
Anti CCP positive			210 (61)
RF positive			221 (65)
DAS28		5.07 (1.2)	
ESR		38 (26)	
CRP, mg/L		37 (38)	
HAQ, 0-3		0.97 (0.61)	
Total Sharp score (SHS)		4.0 (8.2)	
Erosion score (ES)		1.7 (3.8)	
Joint space narrowing score (JSN)		2.3 (5.2)	
Prednisolone			1876 (50)
DMARDS	None		77 (20)
	Methotrexate		155 (41)
	Sulfasalazine		102 (27)
	Other DMARD		44 (12)
	Combination		1 (0)
	Biologics		0 (0)

Anti-CCP, antibodies against cyclic citrullinated peptides; CRP, C-reactive protein; DAS28, Disease Activity Score calculated on 28 joints; DMARDS, disease modifying anti-rheumatic drugs; ESR, erythrocyte sedimentation rate; HAQ, Health Assessment Questionnaire; N, number; RF, rheumatoid factor; SD, standard deviation.

There were no significant differences in baseline characteristics between these 379 patients having and the 460 patients lacking radiographs suitable for DXR-analyses at baseline and one year (data not shown).

### DXR - BMD

**Figure 1:**
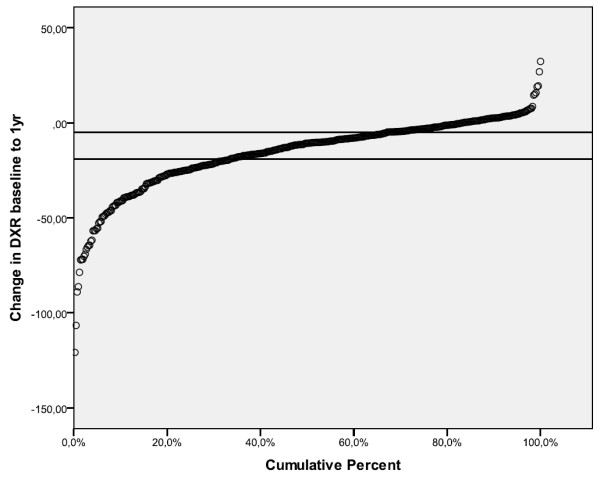
**Cumulative percent plot of the change (mg/cm2) in DXR from baseline to one year**. Reference lines denote tertiles -upper tertile- a change of -18 mg/cm²; lower tertile - a change of -5 mg/cm². DXR, digital X-ray radiogrammetry.

At baseline, the mean (SD) DXR-BMD was 574(94) mg/cm² and the mean (SD) Z-score -0.10 (1.13). The mean (SD) change in DXR-BMD from baseline to one year (ChDXR_1yr_) was -16 (19) mg/cm² (-1.3 mg/cm²/month) and mean (SD) change in Z- score was -0.27(0.35). A cumulative probability plot illustrates ChDXR_1yr _(Figure 1). Figure 1 shows that the lower tertile corresponds to a one year change in BMD of -5 and the upper a change of -18 mg/cm².

### Correlations between decreased DXR-BMD and increased radiographic damage scores

ChDXR_1yr _correlated significantly and inversely with ChSHS at two, five and eight years with the correlation coefficients -0.295, -0.215 and -0.239, respectively, all p < 0.001.

### Hand bone loss was associated with increase in radiographic damage score

The mean ChSHS at two, five and eight years was at all time-points significantly greater in the group of patients with hand bone loss by HBLsdc. The association was most pronounced at two years (Table 2).

**Table 2:** Change in radiographic scores (SHS) in presence or absence of hand bone loss.

	HBLsdc						
		
	No hand bone loss			Hand bone loss			Diff.
Change in SHS	Mean	SD	N	Mean	SD	N	p
ChSHS2yr	4.31	8.36	113	10.81	15.48	242	0.001
ChSHS5yr	11.82	15.36	89	21.49	26.59	199	0.003
ChhSHS8yr	13.31	16.91	81	23.58	28.34	159	0.005

ChSHS, change in SHS; HBLsdc, hand bone loss smallest detectable change; N, number; SD, standard deviation; SHS, Sharp van der Heijde Score; yr, year.

Similarly, the mean ChSHS at these time points was significantly greater in patients with hand bone loss according to HBLtertiles. A *post hoc *pair wise analysis showed that, at two years, the groups of patients with great and moderate HBL had significantly greater ChSHS than the group with no/small HBL while at five and eight years this was the case only for the great HBL group (Table 3).

**Table 3:** Change in radiographic scores (SHS) in patients with great, moderate or no/small hand bone loss.

	HBLtertiles									
	Great bone loss			Moderate bone loss			No/small bone loss			Diff.
	Mean	SD	N	Mean	SD	N	Mean	SD	N	p
Change in SHS										
ChSHS2yr	12.84	18.74	119	8.90	11.33	118	4.44	8.37	118	0.001
ChSHS5yr	23.96	29.64	103	19.03	23.09	91	12.01	15.32	94	0.005
ChSHS8yr	26.04	30.65	81	21.17	26.09	75	13.46	16.74	84	0.011

ChSHS, change in SHS; HBL, hand bone loss; N, number; SD, standard deviation; SHS, Sharp van der Heijde Score; yr, year.

**Figure 2:**
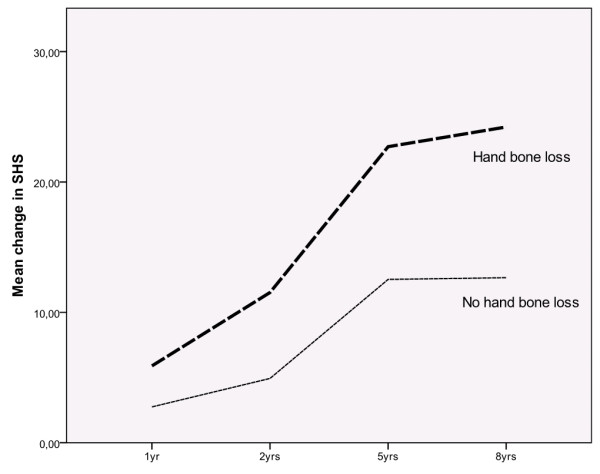
**Radiographic progression, as change in SHS, over eight years in patients with and without HBLsdc**. SHS, Sharpe van der Heijde score.

**Figure 3:**
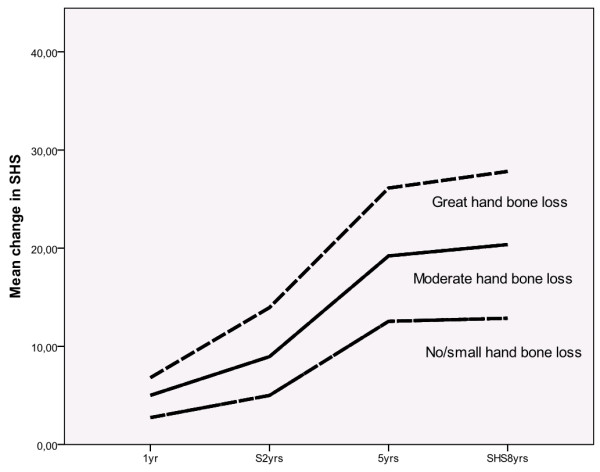
**Radiographic progression, as change in SHS, over eight years in patients with and without HBLtertiles**. SHS, Sharpe van der Heijde score.

The differences in mean ChSHS over time between the groups of patients with HBLsdc and HBLtertiles are illustrated in Figures 2 and 3.

### Hand bone loss was associated with radiographic progression

Radiographic progression from baseline was seen in 25% of the patients after one year, in 41% of the patients at the follow-up visit at two years, in 60% at five years and in 61% at eight years.

At the same time-points 43%, 38%, 37% and 36% of the patients, respectively, still had no erosions. The inter- and intra-observer reliability was 0.94 and 0.99, respectively.

Radiographic progression after two, five and eight years was associated with HBL defined by HBLsdc (Table 4). By HBLtertiles there were significant associations at all time-points except at eight years (Table 5). The association was most pronounced after two years, when 49% of the patients with HBL by HBLsdc had radiographic progression and analyzed by HBLtertiles also 49% (54% of the patients with great and 44% with moderate HBL) had radiographic progression (p < 0.001).

**Table 4:** Radiographic progression in presence or absence of hand bone loss.

	HBLsdc		
	No hand bone loss	Hand bone loss	Diff.
Radiographic progression	N(%)	N(%)	p
2 years	27(24)	118(49)	0.001
5 years	43(48)	129(65)	0.008
8 years	42(52)	105(66)	0.033

HBLsdc, hand bone loss smallest detectable change; N, number.

**Table 5:** Radiographic progression in patients with great, moderate or no/small hand bone loss.

	HBLtertiles			
	Great bone loss	Moderate bone loss	No/small bone loss	Diff.
Radiographic progression	N (%)	N (%)	N (%)	p
2 years	64(54)	52(44)	29(25)	0.001
5 years	72(70)	54(59)	46(49)	0.011
8 years	57(70)	46(61)	44(52)	0.060

HBL, hand bone loss; N, number.

### The performance of DXR-BMD as a predictor of radiographic progression

Univariate analyses by score tests of demographic and clinical variables possibly associated with radiographic progression were performed. Since the predictive ability of a change in DXR-BMD over one year is under study, not only baseline data but also information obtained up to one year after baseline could have useful predictive value and must, therefore, be taken into consideration. The following variables were univariately associated with radiographic progression, at two, five and eight years: ChDXR_1yr_, HBLsdc, HBLtertiles, ChSHS_1yr_, presence of erosions_bl_, anti- CCP as well as number of swollen joints_1yr_; at two years: DAS28_1yr_, GH_1yr_, ESR_bl, _ESR_1yr_, HAQ_1yr _and CRP_1yr _; at five years: DAS28_1yr_, ESR_bl_, ESR_1yr, _CRP _bl_, and CRP_1yr _; and at 8 years: tender joints_bl _and tender joints_1yr _.

Age, disease duration, gender, smoking, baseline disease-modifying anti-rheumatic drug (DMARD) and glucocorticoid treatment as well as baseline DXR-BMD (including Z scores) were not univariately associated with radiographic progression at any time point.

The positive predictive values (PPVs) of the presence of HBL by HBL_sdc _for radiological progression at two, five and eight years (prevalence of 41%, 60% and 61%, respectively) were 49%, 65% and 61%. The corresponding PPVs of anti- CCP were 50%, 73% and 70%; of erosions_bl _56%, 73% and 73%; and of ChSHS1yr (above median) were 76%, 81% and 83%.

ChDXR1yr and the other variables significantly associated with radiographic progression in the univariate analysis were put into multiple logistic regression analyses with radiographic progression at two, five, and eight years as dependent variable. Table 4 shows the two-year result where ChSHS_1yr _and anti-CCP proved to be independent predictors of radiographic progression which also was the result for five years. At eight years ChSHS_1yr, _anti-CCP and the number of tender joints_bl _and tender joints_1yr _were independent predictors.

Since scoring of radiographs is infrequently performed in clinical practice, models excluding radiographic scores (ChSHS_1yr_) were also done. Then ChDXR_1yr _independently predicted radiographic progression at two years in addition to anti- CCP, number of swollen joints_1yr _and presence of erosions_bl _(Table 6). In the prediction models at five and eight years ChDXR_1yr _did not attain statistical significance (p = 0.083 and 0.073, respectively). At five years, erosions_bl_, anti-CCP, number of swollen joints_1yr, _and CRP_1y_, were independent predictors and at eight years anti-CCP, number of swollen joints_1yr _and number of tender joints at baseline and one year.

**Table 6:** Multiple logistic regression of possible independent predictors of radiographic progression at two years.

	Model with x- ray scores^a^				Model without x- ray scores^b^			
	OR	95% CI		p	OR	95% CI		p
Change DXR 1 yr	1.001	.0.974	1.029	.956	0.971	0.955	0.988	.001
Change SHS 1 yr	2.667	2.027	3.511	.000				
Erosions at baseline	.666	.262	1.691	.392	2.592	1.486	4.519	.001
Anti- CCP	3.475	1.332	9.066	.011	3.132	1.701	5.766	.001
Number of swollenjoints 1 yr	1.086	.931	1.267	.296	1.129	1.021	1.248	.018
DAS28 1 yr	1.220	.645	2.307	.541	1.181	.791	1.764	.417
General health 1 yr	.990	.961	1.021	.527	.997	.979	1.016	.761
ESR baseline	.999	.979	1.018	.886	.991	.979	1.004	.178
ESR 1 yr	1.014	.980	1.050	.415	1.005	.982	1.030	.665
HAQ 1 yr	1.327	.570	3.092	.512	1.079	.608	1.916	.795
CRP 1 yr	.974	.929	1.020	.261	1.006	.980	1.032	.656
Constant	.014			.000	.060			.001

^a^R^2 ^= 77%. 91% correct classification. ^b^R^2 ^= 36%. 75% correct classification. Anti-CCP, antibodies against cyclic citrullinated peptides; CI, confidence interval; CRP, C-reactive protein; DAS28, Disease Activity Score calculated on 28 joints; DXR, digital X-ray radiogrammetry; ESR, erythrocyte sedimentation rate; HAQ, Health Assessment Questionnaire; OR, odds ratio; SHS, Sharp van der Heijde Score; yr, year.

HBLsdc and HBLtertiles were univariately associated with radiographic progression at all time-points, but did not significantly contribute to prediction in the multivariate models (data not shown).

The best model predicting radiographic progression was obtained at the two- year follow-up visit. In this model, including radiographic scores Nagelkerke R2 was 77% and 91% of the patients correctly classified while the corresponding figures were 36% and 75% in the model excluding radiographic scores (Table 6). After five years, R2 was 52% and 38% and the proportion of correct classifications was 79% and 75% in the models with and without radiographic scoring, respectively. The corresponding figures for eight years were 44% and 30% and 73% and 72%, respectively.

Replacing ChDXR_1yr _with Z-scores to account for age related bone loss did not improve prediction (data not shown).

### DXR -BMD and clinical outcome

#### Hand bone loss and degree of disease activity after two, five and eight years

Cross tabulation of hand bone loss by HBLsdc and degree of disease activity showed no significant differences in degree of disease activity after two, five and eight years in patients with or without bone loss (data not shown). However, patients with great bone loss by HBLtertiles had significantly high disease activity more often after two years compared with patients with moderate or low activity (16% versus 5% and 6%, respectively, p = 0.009).

#### Hand bone loss and remission

In patients with HBL by HBLsdc, remission was significantly less frequent after two years than in patients without (38% versus 49%, p = 0.037) but not after five (43% versus 39%, p = 0.49) or eight years (40% versus 48%, p = 0.24).

In patients with great bone loss by HBLtertiles, remission was less frequent after two years than in patients with moderate or no/small bone loss (34% versus 41% and 50%, respectively, p = 0.045). No significant differences were seen after five years (42% versus 46% and 39%, respectively, p = 0.59) or eight years (36% versus 46% and 47%, respectively, p = 0.27.)

#### Hand bone loss as a predictor of remission aftertwo, five and eight years

Although DXR-BMD showed a univariate association with remission at two and eight years, neither bone loss by HBLsdc or HBLtertiles nor by ChDXR_1yr _was an independent predictor of remission after two, five and eight years in models including and excluding x-ray scores (data not shown).

## Discussion

In this study on 379 patients with early RA who were followed for eight years we have studied the potential of HBL during the first year measured by DXR to predict radiographic joint damage progression after two, five and eight years.

In accordance with previous studies, HBL above SDC was significantly associated with radiographic joint damage. This was the case also when HBL was defined by tertiles as great, moderate or no/small.

In the early stage of the disease the patients had bone loss more often than they had radiographic progression. Thus, after one year HBL was present in 68% of the patients while progressive joint damage was seen in only 25%. With time radiographic progression increased to more than 60%, which is in agreement with the report by Güler-Yüksel *et al. *[26], who showed bone loss after one year in 68%, progressive joint damage in 18% and after four years progressive joint damage in 30%. The explanation for this difference is that localized loss of BMD predates erosions and joint destruction [27,28].

Change in DXR-BMD between baseline and one year correlated significantly and inversely with change in SHS at all time-points. This is in agreement with previous studies [13,14,29]. In an observational study, Stewart *et al. *showed that measurement of HBL over one year, using DXR, correlated with erosive changes in patients with early RA at four years follow-up [29] and Hoff *et al. *also showed that patients with hand BMD loss at one year had more radiographic damage at five and ten years in comparison with patients without HBL [30]. However, in these studies, the correlations between hand BMD and joint damage were found to be small suggesting that the predictive ability of HBL for joint damage may be limited. This notion is further supported by the modest PPVs of HBL for joint damage found in the present study.

The change in SHS from baseline to one year and the presence of anti-CCP, but not change in DXR-BMD, were the only independent predictors of radiographic progression at two years. This is in line with the BeSt study reporting that hand BMD loss as a risk factor for further radiographic damage is largely overridden by change in SHS measured over one year [26]. This implies that the first year change in DXR-BMD does not add to the therapeutic decision if radiographic scores are available. However, in clinical practice, scoring radiographs is not frequently performed, as it is a time-consuming method requiring special training.

Therefore, in the absence of radiographic scores, DXR-BMD, which today may be easily accessible in clinical practice, might be a helpful predictor. Indeed, the present data show that in the absence of radiographic scoring the change in DXR-BMD was an independent predictor of radiographic progression, significant at two years, but not at five and eight years.

In the present study, change in SHS over the first year was the single best predictor of further radiological progression. This predictor attained the highest PPVs and contributed highly to create the best regression model for predicting radiological outcome at two years, in which also the presence or absence of baseline erosions, anti-CCP and number of swollen joints after one year were independent predictors. By this model Nagelkerke R^2 ^was 77% and 91% of the patients could be correctly classified. Thus, change in SHS over the first year is indeed a promising predictor for use at this stage of the disease. However, it would be ideal if reliable predictors could be identified at an earlier stage of the disease.

Ongoing studies including measuring changes in DXR-BMD over shorter time periods, for example, three and six months, are awaited with great interest. Recently a study with six months data on hand BMD loss was published [31]. Here DXR-BMD loss was measured from baseline to six months in 80 patients with early (mean disease duration 11 month) undifferentiated arthritis. They found that great BMD loss was associated with RA development after one year. It should be noted that the cut-off value for 'great bone loss' was considerably higher than that used in the present study (≥2.5 versus 1.5 mg/cm²/month).

It has been suggested that treatment with biologics may decelerate the development of erosions more than it reduces HBL [32]. However, since the present study was performed before the introduction of biologics, this issue could not be further addressed.

Loss of bone measured by HBLsdc and HBLtertiles was more frequent in patients with high disease activity and in patients not in remission after two years but not in later phases of the disease. HBL did not come out as an independent predictor of clinical outcome in multiple regression analyses. In 145 patients from the BeSt study, where change in hand BMD by DXR was investigated, an increase in BMD occurred mostly in patients with continuous remission by the EULAR criterion [33]. However, continuously low disease activity did not appear to provide less loss in hand BMD than continuously high disease activity. Therefore, although reduction of BMD is a consequence of inflammation, DXR-BMD is not a suitable tool for predicting disease activity in the future.

A shortcoming of this study was that, at several sites, the technical conditions at acquisition of X-rays were such that BMD could not be measured for some patients which led to the loss of several patients from the study, but the included patients did not differ in baseline characteristics from those not included. Furthermore, the patients with early RA in this study fulfilled the ACR 1987 criteria, whereas patients with very early RA who presented with limited clinical symptoms were not included until they fulfilled the criteria. This means that we have no data, neither of radiographic damage nor of bone mineral density from the very early phase of RA.

## Conclusions

To conclude, the present study confirms previous studies reporting an association of change in DXR-BMD over the first year of RA disease with progression of radiographic joint damage over an extended period of time. However, the change in DXR-BMD was not an independent predictor of radiographic joint damage progression. Instead, change in SHS from baseline to one year was a strong predictor of radiographic progression both after two years and after longer follow-up visits at five and eight years. On the other hand, when radiological findings were excluded the association was independent. This suggests a possible predictive role of DXR-BMD in clinical practice when radiographic evaluation is not available. However, since the association was significant only after the follow-up at two years and the PPVs of bone loss for radiological progression were quite small, further studies are required before this can be established. Further studies on DXR may focus on the diagnosis of early RA also considering sex- and age-related DXR data that define a threshold value to discriminate possible patients with RA. In particular, studies with a shorter interval between measurements of DXR-BMD are encouraged to evaluate its predictive power in early RA, ideally before joint damage has become identified radiographically.

## Abbreviations

ACR: American College of Rheumatology; Anti-CCP: anti-cyclic citrullinated proteins; BARFOT: Better Anti-Rheumatic FarmacoTherapy; BMD: bone mineral density; ChDXR: change in DXR; ChSHS: change in SHS; CRP: C-reactive protein; DAS: Disease Activity Score; DXR: digital X-ray radiogrammetry; E: erosions; ESR: erythrocyte sedimentation rate; EULAR: European League against Rheumatism; GH: general health; HAQ: Health Assessment Questionnaire; HBL: hand bone loss; ICC: intra-class correlation coefficient; JSN: joint space narrowing; PPV: positive predictive value; RA: rheumatoid arthritis; RF: rheumatoid factor; sdc: smallest detectable change; SHS: Sharp van der Heijde Score; VAS: visual analogue scale.

## Competing interests

JK is an employee of Sectra Imtec AB. KF has received honoraria from Abbott and Bristol-Myers Squibb, not related to this study. All other authors declare they have no competing interests.

## Authors' contributions

All authors contributed to the study design, acquisition of data, analysis and interpretation of data, and manuscript preparation. BS contributed with statistical analysis. All authors read and approved the final manuscript.
